# Adaptive, platform trials assessing therapies for hospitalized COVID-19 patients: Informed consent forms omitted a few important elements of information

**DOI:** 10.7189/jogh.13.06019

**Published:** 2023-05-12

**Authors:** Rafael Dal-Ré, Teck Chuan Voo, Søren Holm

**Affiliations:** 1 Epidemiology Unit, Health Research Institute-Fundación Jiménez Díaz University Hospital, Universidad Autónoma de Madrid, Madrid, Spain; 2 Centre for Biomedical Ethics, Yong Loo Lin School of Medicine, National University of Singapore, Singapore; 3 Centre for Social Ethics and Policy, Department of Law, School of Social Sciences, University of Manchester, University of Manchester, UK; 4 Center for Medical Ethics, HELSAM, Faculty of Medicine, University of Oslo, Norway

## Abstract

**Background:**

The information provided to participants of adaptive platform trials assessing therapies for COVID-19 inpatients is unknown. We aim to evaluate it by reviewing participant information sheets/informed consent forms (PIS/ICFs).

**Methods:**

We searched the Cochrane COVID-19 Study Register and ClinicalTrials.gov (28 March 2022) to identify non-industry-sponsored adaptive platform phase 2+ trials with publicly available protocols and PIS/ICFs, selecting versions closest to the initial one. We assessed the elements of information included in the Good Clinical Practice guidelines and the Declaration of Helsinki as present, absent, or deficient (incompletely described).

**Results:**

We included PIS/ICFs of 11 trials (ACCORD-2, ACTIV-1IM, Bari-SolidAct, CATALYST, Discovery, HEAL-COVID, ITAC, RECOVERY, REMAP-COVID, Solidarity and TACTIC-R), which were 4-32 pages long (median (md) = 11). Between two and 11 (md = 6) of the 25 different elements of information assessed were omitted or deficiently described in the PIS/ICFs of the 11 trials. Information about providing trial results, investigators’ conflicts of interest, post-study provisions, payment to and anticipated expenses for participants, number of participants, and on whether participants will receive new information that could impact their decision on staying in the trial, were omitted or deficiently described in at least five PIS/ICFs.

**Conclusions:**

Investigators failed to include a few important elements of information in the trial’s PIS/ICF deemed relevant by international standards. In protocols of future trials, investigators should explain why elements of information specified in the Good Clinical Practice guidelines and/or by the Declaration of Helsinki were omitted from the PIS/ICFs.

Several studies have assessed the scientific quality of clinical trials conducted to assess medicinal products for the treatment of COVID-19 patients, both in terms of methodology [1-3] and reporting [4]. However, less attention had been paid to their ethical quality, despite the availability of specific normative documents on how research may be ethically conducted in global health emergencies [5,6]. Research on the informed consent process of COVID-19 clinical trial participants has been scarce and, to our knowledge, limited to vaccine trials. Some reports have highlighted that participants’ information sheets were too long and difficult to read for healthy individuals participating in COVID-19 vaccine trials [7,8].

Concerning hospitalised COVID-19 patients, it should be acknowledged that the pandemic, due to imposed physical distancing measures and increasing health professional workload and stress, has immensely complicated the process of seeking informed consent from potential trial participants. In hospital wards, clinical researchers faced problems when trying to enroll patients in a clinical trial, ranging from risking infection to the likely incapacitation of many patients and the need to find legally authorised representatives [9]. Following guidance from the European Medicines Agency [10] and the USA Food and Drug Administration [11], research ethics committees (RECs) have permitted e-consent, video, and telephone consent. Nevertheless, transitioning to electronic documentation of informed consent, despite protecting investigators from contracting SARS-CoV-2, was difficult in many settings [9].

The Declaration of Helsinki [12] and the Good Clinical Practice guidelines (GCP) [13] describe the elements of informed consent that should be included in each trial participant’s information sheet/informed consent form (PIS/ICF); they form the basis for discussion between potential participants and investigators. As prior studies have highlighted major problems with the information provided to participants in COVID-19 vaccine trials (for example, only one of four PIS/ICFs from phase 3 trials mentioned that the control (placebo) group might receive vaccine) [7], we aimed to review and evaluate the completeness of the information provided in the PIS/ICF of clinical trials assessing COVID-19 therapies. We focused on adaptive, platform randomised controlled trials (adRCTs) that study “multiple targeted therapies in the context of a single disease in a perpetual manner, with therapies allowed to enter or leave the platform on the basis of a decision algorithm” [14]. Specifically, we focused on hospitalised patients since, as mentioned above, the consent process had specific hurdles to solve in this setting and because some of these adRCTs have been (and remain) critical for the rapid generation of efficacy results on a number of medicinal products for supporting their acceptance (or rejection) by scientific and clinical communities [15,16]. These trials are also usually large and generally well-funded, so it is reasonable to expect their PIS/ICF should meet acceptable standards by providing relevant and important information.

## METHODS

We conducted a search on 28 March 2022, aiming to include non-industry-sponsored, self-labelled adRCTs assessing therapies for hospitalised COVID-19 patients. These trials should have made the protocol and PIS/ICF publicly and freely available on ClinicalTrials.gov, on their website, or as supplements to published articles. We sought document versions closest to the initial ones. When the protocol was available, but the PIS/ICF was not, we sent an e-mail to the contact investigator/corresponding author/trial website requesting it. Ideally, trials should have results posted on trial registers and/or published in peer-reviewed journals.

Since the Cytel COVID-19 Clinical Trials Tracker [17] was not operative, we first reviewed the Cochrane COVID-19 Study Register for phase 2+ adRCTs assessing therapies for hospitalised COVID-19 patients, followed by ClinicalTrials.gov. Finally, we reviewed all adRCTs included in recent reviews of this topic [18,19] for those adRCTs meeting our criteria. The searches are shown in Table S1 in the **Online Supplementary Document**.

The trials fulfilling our search criteria were: ACCORD-2, ACTIV (1 IM, 3, 4A, 4HT & 5 (BET-A, BET-B and BET-C)), CATALYST, Discovery, EU-SolidAct (Bari-SolidAct), HEAL-Covid, ITAC, RECOVERY, REMAP-COVID, Solidarity (Solidarity and Solidarity Plus) and TACTIC-R. The PIS/ICF from ACCORD-2, Bari-SolidAct, Discovery and REMAP-COVID, were requested up to three times between late March and late April, and we received positive responses from ACCORD-2, Bari-SolidAct and Discovery. Since REMAP-COVID did not share its master PIS/ICF with us, we used the latest publicly available version posted on ICNARC [20] which provided the PIS/ICF for UK patients. If the same organization conducted two or more trials, we only included the first one in our assessment (i.e. ACTIV and Solidarity).

Two researchers (RDR and TCV) independently reviewed the PIS/ICF of the 11 included adRCTs, assessing the presence of the 20 elements of information specified in the GCP and the 10 mentioned in the Declaration of Helsinki (Table S2 in the **Online Supplementary Document**). Five elements (aims, methods, benefits, risks, voluntariness) are common to both the GCP and the Declaration of Helsinki, so we assessed 25 different elements of information as “yes” (i.e. appropriately described), “no” (i.e. absent) or “deficient” (i.e. incompletely described). Discrepancies were resolved by discussion with the third author (SH).

Finally, we searched for published trial results in PubMed, the trial’s website, or the register in which it was registered. If more than one publication was found, we only checked the initial one to see if the authors disclosed conflicts of interest with the pharmaceutical industry.

## RESULTS

We included 11 adRCTs: ACCORD-2, ACTIV-1IM, Bari-SolidAct, CATALYST, Discovery, HEAL-COVID, ITAC, RECOVERY, REMAP-COVID, Solidarity and TACTIC-R (**Table 1**). These adRCTS were sponsored by organizations/institutions from the USA (ACTIV-1IM and ITAC), France (Discovery), The Netherlands (REMAP-COVID), Norway (Bari-SolidAct), the World Health Organization (Solidarity) and the UK (the remaining five). All were funded by public institutions, except ACCORD-2 and TACTIC-R, which were funded by pharmaceutical companies. They were conducted in a single country, except Bari-SolidAct, Discovery, ITAC, REMAP-COVID, and Solidarity. All assessed therapies administered exclusively at hospitals, except for HEAL-COVID, which assesed the effectiveness of long-term (one year) medicines to improve longer-term clinical outcomes from COVID-19.

**Table 1:** Information explicitly mentioned in the trial protocols of the 11 COVID-19 adaptive, platform trials included in this study, and the references where trial results were published

	Adaptive, platform clinical trial (protocol version)										
	ACCORD-2-2 (April 2020)	ACTIV1 (1.0, September 2020)	Bari SolidAct (Inter. Specific Appendix 2.0, November 2021)	CATALYST (BMJ Open 2021*)	Discovery (7.0, April 2020)	HEAL-COVID (5.0, August 2021)	ITAC (1.0, August 2020)	RECOVERY (4.0, April 2020)	REMAP-COVID (1.0, March 2020)	Solidarity (10.0, March 2020)	TACTIC─R (2.0, May 2020)
**Trial conducted following**											
Declaration of Helsinki	Yes	No	Yes	Yes	No	Yes	Yes	No	Yes	No	Yes
GCP†	Yes	Yes	Yes	Yes	Yes	Yes	Yes	Yes	Yes	Yes	Yes
**Trial features**											
Sample size	1800	2160	950	180	3100	2631	500	‘thousands’	6800	‘several thousands’	687 to 1167
Masking	Open	Double-blind	Double-blind	Open	Open	Open	Double-blind	Open	Open	Open	Open
Participating country (-ies)	UK	USA	Austria, Belgium, France, Germany, Ireland, Italy, Luxembourg, Norway, Portugal, Spain	UK	France, Luxembourg	UK	Argentina, Denmark, Germany, Greece, Indonesia, Israel, Japan, Nigeria, Spain, UK, USA	UK	Australia, Canada, France, Ireland, the Netherlands, New Zealand, UK, USA	30 countries in 4 continents	UK
Sponsor‡	University Hospital Southampton, UK	Dr Daniel Benjamin, Duke University, USA	Oslo Univ. Hospital, Norway	University Birmingham, UK	INSERM, France	Cambridge University Hospitals, UK	University Minnesota, USA	University of Oxford, UK	UMC Utrecht, The Netherlands	World Health Organization, Switzerland	Cambridge University Hospitals, UK
**Consent fatures**											
From participant’s relative or LAR	Yes	Yes	Yes§	Yes‖	Yes	Yes‖	Yes	Yes‖	Yes‖	Yes	Yes‖
Deferred	Yes	Yes	Yes	Yes	No	Yes	Yes	Yes	Yes	Yes	Yes
Written	Yes	Yes	Yes	No¶	Yes	Yes	Yes	No¶	Yes	Yes	Yes
Verbal	No	No	Yes**	No	No	No	No	No	No††	No	No
Other	LAR (Phone & email)	No	Witness (Phone or web video)	LAR (email)	No	LAR (Phone & written)	No	No	LAR (Phone & email)	No	No
**Results published**											
Reference	NA	NA	Trøseid et al [21]	Fisher et al [22]	Ader et al [23]	NA	ITAC Study Group [24]	RECOVERY Collab. Group [25]	Writing Com. REMAP-CAP Investigators [26]	WHO Solidarity Trial Consor. [27]	NA

GCP – Good clinical practice, LAR – legal authorized representative, NA – not applicable

*Veenith, et al [39]

†Good clinical practice is an international ethical and scientific quality standard for designing, conducting, recording, and reporting trials that involve the participation of human subjects. Clinical trials should be conducted in accordance with the ethical principles that have their origin in the Declaration of Helsinki (2.1).

‡ Information provided by trial register.

§Consent provided by an independent clinician is accepted.

‖Consent provided by an independent clinician is accepted, according to the requirements of the UK Health Research Authority.

¶The informed consent form explicitly showed that consent must be given in writing.

**If national regulations allow, verbal consent in these circumstances.

††Verbal consent was acceptable in the first published article [25].

Since all adRCTs were conducted in accordance with the GCP, they had to follow the provisions of the Declaration of Helsinki, which was explicitly mentioned in all but ACTIV-1IM, Discovery, RECOVERY, and Solidarity trial protocols. All trial protocols explicitly mentioned that informed consent could be provided by participants or their legal authorised representatives (or relatives), or that deferred consent could be acceptable from participants incapable of consenting. The Discovery protocol did not mention the need for deferred consent when the legal representative provided the informed consent. Seeking written informed consent was the norm, although Bari-SolidAct accepted verbal and witnessed consent by phone or web video, if legally accepted by a country regulation.

**Table 2** shows the elements of informed consent specified by the GCP and the Declaration of Helsinki that were omitted from the PIS/ICF of one or more of the 11 adRCTs assessed. Eleven of the 20 elements of informed consent specified by the GCP,

**Table 2:** Elements of informed consent to be included in the written informed consent form following the guidelines for good clinical practice (GCP) and the Declaration of Helsinki. Only those elements that were not (or were deficiently) included in one or more of the informed consent forms of the 11 adaptive, platform clinical trials assessed are shown. (Table S2 in the **Online Supplementary Document** provides the actual wording of the elements of informed consent included in both the good clinical practice guidelines and the Declaration of Helsinki)*†

Elements of information	Adaptive, platform clinical trial (ID)										
	ACCORD-2 (ISRCTN57085639)	ACTIV-1IM (NCT04593940)	Bari SolidAct (NCT04891133)	CATALYST (ISRCTN40580903)	Discovery (NCT04315948)	HEAL-COVID (ISRCTN15851697)	ITAC (NCT04546581)	RECOVERY (NCT04381936)	REMAP-COVID (NCT02735707)	Solidarity (ISRCTN83971151)	TACTIC-R (NCT04390464)
PIS/ICF version	1.0, April 2020	2.0, December 2020	3.0, March 2022	5.0, September 2020	1.0, April 2020	4.0, August 2021	September 2020	4.0, April 2020	1.11, November 2021‡	10.0, March 2020	1.2, May 2020
No. of pages	17	32	8	9	11	7	13	4	14	4	12
PIS/ICF copy given to participant	Yes	Yes	No	Yes	Yes	Yes	Yes	Yes	Yes	Yes	Yes
**Good clinical practice (4.8.10)**											
d. Clinical trial procedures	Yes	Yes	Yes	Yes	Yes	Yes	Yes	Yes	Yes	No	Yes
g. Risks and inconveniences	Yes	Yes	Yes	Yes	Yes	Yes	Yes	Deficient§	Yes	Yes	Yes
j. Compensation or treatment available for any injury	Yes	Yes	Yes‖	Yes	Yes	Yes§	Yes	Yes‖	Yes‖	No	Yes
k. Prorated payment	No	Yes	No	No	No	No	Yes	Yes	No	Yes	Yes
l. Anticipated expenses	No	Yes	No	Yes	No	Yes	Yes	Yes	No	No	Yes
n. Monitor, auditor, RECs, regulators, have access to participants’ medical records	Yes	Yes	Yes	Yes	Yes	Yes	Yes	Yes	Yes	No	Yes
p. Providing new information available	Yes	Yes	No	No	No	Yes	Yes	Yes	No	No	Yes
q. Contact person for information and injury	Yes	yes	Deficient¶	Yes	Yes	Yes	Yes	Yes	Yes	Deficient**	Yes
r. Reasons for stop participation	Yes	Yes	Yes	No	Yes	Yes	Yes	No	Yes	Yes	Yes
s. Duration in the trial	Yes	Yes	Yes	Yes	Yes	Yes	Yes	No	Yes	Yes	Yes
t. Number of participants	Yes	Yes	No	No	No	Yes	Yes	No	Yes	No	Yes
**Declaration of Helsinki**											
Sources of funding	No	Yes	Yes	Yes	No	Yes	Yes	Yes	Yes	Deficient††	Yes
Possible CoI	No	Yes	No	No	No	Deficient‖‖	Deficient¶¶	Deficient‖‖	Deficient***	Deficient‖‖	No
Poststudy provisions	Yes	No	No	No	Yes	Yes	No	No	No	No	Yes
Inform trial results	Deficient†††	Deficient‡‡‡	Deficient§§§	Deficient‖‖‖	Deficient†††	Deficient¶¶¶	Deficient‡‡‡	No****	Deficient††††	Deficient‡‡‡‡	Deficient§§§§

CoI – conflicts of interest, PIS/ICF – Participants’ information sheet and informed consent form, REC – research ethics committee

*Participants’ information sheet is included as part of the informed consent form.

†All the elements of informed consent included in the guidelines for good clinical practice E6 (R2) and the Declaration of Helsinki are shown Table S2 in the **Online Supplementary Document**.

‡The only one available for COVID-19 patients was posted on ICNARC [20] which provided the PIS/ICF for UK patients.

§The side effects associated with the medicinal products assessed was deficiently described in this PIS/ICF; however, this approach has been modified and now a limited number of the side effects are mentioned (Table S4 in the **Online Supplementary Document**).

‖Participants are informed of the existence of insurance.

¶Contact provided for requesting more information but not in case of injury.

**Contact provided in case of injury but not for requesting more information.

††WHO is mentioned as co-sponsor: this could be regarded as a funder by participants as no specific funder was provided.

‖‖Participants were only informed that investigators will not be paid.

¶¶Participants were informed that the samples will not be sold and that they will not be used for profit-making.

***Participants were informed that the pharma companies are not involved in the design, analysis, or reporting of results from the trial.

†††Participants were asked to talk to their doctor to have access to the results.

‡‡‡Participants were informed that Clinicaltrials.gov will include a summary of the results, and that they can access them at any time.

§§§Participants were informed that they have the right to access the results of the study.

‖‖‖Participants were informed that at the end of the trial, the findings will be published in peer-reviewed medical and scientific journals, and that these publications will be available upon request from the trial doctor, and that a lay summary of the results will be available on the trial websites.

¶¶¶As described in the PIS/ICF, participants were requested to inform whether they would like to receive the results at the end of the study.

****However, participants did receive a letter informing them of the results.

††††Participants were informed that the results, once the trial has completed, will be posted on the trial website.

‡‡‡‡Participants were informed that the findings will be freely available, but not when, how and where to find them.

§§§§Participants were informed that they can receive a copy of published results by contacting their trial doctor.

and four of the 10 mentioned in the Declaration of Helsinki were omitted or assessed as deficient in one or more of the PIS/ICFs.

Among the GCP elements, information on the clinical trial procedures, possibility of access to participant data for monitors, auditors, members of RECs, and regulatory agencies, compensation, and the existence of available treatment for any injury caused by the trial were only omitted in Solidarity’s PIS/ICF. Information on payment to participants was omitted in six adRCTs.

Information on the number of participants to be recruited, the anticipated expenses that participants could incur, and new information that could impact their decision on staying in the trial were omitted in the PIS/ICF of five adRCTs. Notably, only the PIS/ICF of the RECOVERY trial virtually did not mention any risks or adverse effects of the assessed medicinal products.

Among the elements of information mentioned in the Declaration of Helsinki, source of funding was only omitted from the ACCORD-2 and Discovery PIS/ICFs. However, information on possible conflicts of interest and participants’ access to trial results were omitted or insufficiently described in almost all but one (ACTIV-1IM) adRCT.

We found significant differences in length among the PIS/ICFs of the adRCTs assessed, ranging from four pages (RECOVERY, Solidarity) to 32 pages (ACTIV-1IM) (median (md) = 11). Considering the 25 different elements of information assessed, Solidarity PIS/ICF had the highest number of omitted (n = 7) or deficiently informed (n = 4) elements, while ACTIV-1 IM and TACTIC-R had the lowest number of omitted (n = 1) or deficiently reported (n = 1) elements. All PIS/ICF except that of the Bari-SolidACt stated that participants will receive a copy of the document.

Seven adRCTs published the results. A variable number of authors of all articles disclosed conflicts of interest with various companies outside the submitted work (Table S3 in the **Online Supplementary Document**). Several authors of trials administering Gilead’s remdesivir (Bari-SolidAct, Discovery, ITAC, Solidarity) disclosed conflicts of interest with this company.

## DISCUSSION

The use of the adRCT design increased during COVID-19 pandemic [19], making assessments of how they have been and are being conducted important, especially as most COVID-19 trials were small trials, assessing similar therapies in similar populations, with high probability of risk of bias and with an expected low level of evidence [1,3,28].

To our knowledge, this is the first analysis of the elements of information included in the PIS/ICF of adRCTs assessing therapies for COVID-19 inpatients. We found that several elements were deficiently or incompletely described or even omitted which could have otherwise been of critical importance to (potential) trial participants. However, they could also be less relevant or salient when considering the patient population of these trials. The large variations in the length of these documents (up to eight times among the analysed PIS/ICFs) may partly be due to different approaches to information taken by investigators, and partly due to different regulatory requirements imposed by RECs and other regulatory bodies. For those adRCTs that were to be conducted in several countries, the definitive PIS/ICF used in each country could have been edited following national clinical trial regulations, as recently shown in a large COVID-19 adRCT [29]. This could partially explain – but not justify – why, for example, the Solidarity PIS/ICF was the one with more deficiencies and omissions. We fully understand the tension between the urgency of starting to recruit participants in these adRCTs aimed to respond to important treatment questions in hospitalised patients during a pandemic and the detailed disclosure of those elements of information regarded to be relevant to appropriately informed potential participants.

### Important elements of information

One unexpected finding was an almost complete lack of information on the side effects of the assessed medicines in RECOVERY’s PIS/ICF, which have been gradually modified. In the last reviewed PIS/ICF (v22.0, 5 March 2022), a limited number of side effects are mentioned in relation to each medicine (Table S4 in the **Online Supplementary Document**). The amount of provided information on side effects considered appropriate by investigators and RECs can vary substantially. For example, the 5 March 2022 RECOVERY PIS/ICF provided 169 words on seven medicines, while the ACTIV-1IM provided 1189 words on four medicines. A prudent approach for information on the side effects of medicines assessed in trials involving severely ill patients (and in which legal representatives are commonly involved) could be the one taken in Solidarity: 291 words for four medicines. While not the only aspect, the number of words is crucial as long texts could hamper comprehension.

The second relevant element of information that was poorly disclosed in PIS/ICFs concerns informing participants about the trial results. RECOVERY participants were sent a letter on the trial results, despite being informed that they will not receive one, suggesting that the decision of informing participants about the results was likely made after the trial started. HEAL-COVID participants were asked to decide whether they would like to receive the results at the end of the study. The information provided in the remaining nine adRCTS was clearly deficient, since getting the results would require skills or knowledge that not all participants have or a proactive action from participants. In ACTIV-1IM and ITAC, participants were informed that ClinicalTrials.gov will include a summary of the results, accessible at any time. While ITAC has posted the results on ClinicalTrials.gov, ACTIV-1IM has yet to do so. CATALYST informed participants that they could request the published articles from their doctor and that a lay summary will be available on the trial website. TACTIC-R participants could access the published results by contacting their trial doctors. Participants in Bari-SolidAct were informed that they have right of access to the study results, but no specific information was given on how they could exercise it. REMAP-COVID informed participants that results will be available on the trial website, which they now are. ACCORD-2 and Discovery participants were informed that they should contact their doctors to access the trial results, with no further details. Finally, participants in Solidarity were informed that the findings will be freely available, but not when, how, and where – unless this was included at a country-level PIS/ICF.

Informing trial participants of the aggregate results, required by the Declaration of Helsinki since 2013 [12], is not yet a common practice. A recent survey among investigators showed that fewer than half had done it or were planning to do it [30]. Several studies have identified what and how to communicate trial results [31]. Investigators should proactively inform each participant about trial results in writing and in lay language, even if such processes were time-consuming. However, this was practiced by only two (CATALYST and RECOVERY) of the 11 adRCTs included in this study. Asking participants to read scientific reports or summaries on the trials websites denotes little interest to them as key factors in conducting the trial. If RECOVERY investigators, who have enrolled more than 47 000 participants, were able to send a two-page letter to all of them, thanking for their participation and informing them about the trial results [32], this approach might reasonably be adopted by all trials.

The third poorly described element of information included in the Declaration of Helsinki was the disclosure of conflicting interests, omitted by PIS/ICFs of five adRCTs, with three more adRCTs only mentioning that physicians were not paid for participating in the trial, overlooking that conflicts of interest have a much broader scope than mere financial gain [33]. Some authors of the seven articles reporting trial results included in this analysis have disclosed conflicts of interest with industry. It is unfair that article readers were informed about this, but potential participants at the time of consenting were not. What is relevant to a participant is the existence of possible conflicts of interest of investigators in the site where the participant was invited to participate. The ascertainment of conflicts of interest can be difficult in trials in which the drugs under investigation are changed from time to time (e.g. RECOVERY, REMAP-COVID), but this should not be an obstacle to inform trial participants. The most efficient method of achieving this must be agreed with the relevant REC.

ACTIV-1IM was the only trial that broadly addressed this (**Table 3**), although other investigators might regard their approach as too detailed or unsuitable for their trial. Occasionally, an adRCT may be strongly supported by relevant health authorities, which could create conflicts between the treating health care professionals in their roles as clinicians and as investigators of the adRCT (e.g. see letter from the Chief Medical Officers of the UK nations in relation to RECOVERY) [32]. Adequately disclosing investigators' conflicts of interest to trial participants could be a difficult goal to achieve, considering that many researchers report this information incorrectly even in peer-reviewed publications [34]; however, it is nevertheless necessary.

**Table 3:** Examples of wordings which were used to inform trial participants on conflicts of interest and post-study provisions

Conflicts of interest information	
Trial: ACTIV 1IM. PIS/ICF: version 2.0, 31 December 2020	The policy of the NIH is to evaluate investigators at least yearly for any conflicts of interest. Research participants may review the system for assessing conflicts of interest by checking the web site link: http://ethics.od.nih.gov/forms/Protocol-Review-Guide.pdf. Copies of the standards may also be requested by research participants. No NIH investigator involved in this study receives payments or other benefits from any company whose drug, product or device is being tested. This study has investigators that are NIH employees and some that are not. All non-NIH investigators are required to follow the principles of the Protocol Review Guide but are not required to report their financial holdings to the NIH.
**Poststudy provisions**	
Trial: TACTIC-R. PIS/ICF: version 1.2, 4 May 2020	What happens when the trial stops? Once the trial has ended you will be referred back to regular treatments. Pending the results of the trial, treatment guidelines may change

NIH – National Institutes of Health, PIS/ICF – Participant’s information sheet/Informed consent form

### Not so important elements of information

There are three poorly described elements of information that some RECs could have regarded as lacking relevance to trial participants. First, providing new information that might cause participants to change the decision on staying in the trial; since experimental therapies were given for a limited number of days in all these trials (except HEAL-COVID), it is unlikely that new information will appear while participants are still receiving these medications. This approach, however, is not applicable to participants of control groups that could change their minds at any time. Besides HEAL-COVID, five other adRCTs informed participants on this subject. Second, all but four adRCTs omitted statements on informing participants about post-study provisions. However, TACTIC-R provided this information in two short sentences (**Table 3**), so it seems that this information could have been included in all adRCTs, of most relevance to participants in trials conducted in countries with no universal health coverage. Third, the number of participants that was omitted in the PIS/ICF of five trials.

### Limitations

One limitation of our analysis is that we did not conduct a formal systematic search of all COVID-19 adRCTs inpatients although we searched the Cochrane COVID-19 Study Register (among others) that is filled from the most important databases (Table S1 in the **Online Supplementary Document**). However, we aimed only to review the PIS/ICF of typical and relevant trials. Unfortunately, we could not access the first version of the PIS/ICF used to recruit REMAP-COVID participants. Six of the other 10 original PIS/ICFs belong to trials conducted in a single country (the UK or the USA), so they are not expected to have undergone changes in any of the participating sites. However, edits to their original PIS/ICF should be expected in the other four trials in more than one country. This could be especially relevant in trials like Solidarity and ITAC, conducted in countries on four continents. Future studies should assess possible differences in PIS/ICF between countries participating in the same trial after being evaluated by the relevant RECs of each country [29]. The limited number of PIS/ICFs assessed also limits the generalisation of our findings to other adRCTs conducted in COVID-19 inpatients. Additionally, adRCTs encompass only a minority of trial designs used to assess therapies for hospitalised COVID-19 patients. The fact that some of the PIS/ICF referred to the simultaneous assessment of several medicinal products (e.g. RECOVERY, Solidarity) might have influenced the approach taken by investigators when writing these documents and relevant RECs when reviewing them. Where only one experimental intervention was assessed (e.g. Bari-SolidAct, ITAC), the PIS/ICF could be expected to be similar to those in traditional RCTs.

## CONCLUSIONS

Considering recent proposals to reshape the informed consent process [35], providing the right content and amount of written information to trial participants (or their legal representatives) remains crucial in allowing them to make an informed decision, especially as a copy of the PIS/ICF is provided to participants so it can be checked and referred to later – of special importance with deferred consent. The amount of information can vary between trials. In a given trial, the differences in the elements of information included in the PIS/ICF should be minimised, although the amount provided per element could vary depending on the different countries and national regulation requirements. This can also impact on the number of elements of information included in the PIS/ICF which can differ in countries conducting the same trial and having the same original document provided by the trial sponsor [29]. Even if investigators design a streamlined study aiming to minimally disrupt the usual clinical service, research ethics requires that potential participants be adequately informed. The GCP and the Declaration of Helsinki set the standard elements of information that should be provided in writing. Any deviation from this standard – even considering the challenges of conducting research in response to a pandemic emergency [6] – should be based on strong, scientifically- and ethically-sound reasons. It is not easy to correctly balance between the amount of information to provide and the clarity in how it is presented, to prevent overwhelming participants, especially when dealing with hospitalised or critically ill patients, like those recruited in these adRCTs. Lessons learned from the COVID-19 pandemic should help set a future informed consent process research agenda for future pandemics [36].

RECs have faced unprecedented challenges to accelerate review of COVID-19 research, including with trial protocols [37,38], but the perceived quality of the review process did not seem to be negatively impacted in some countries [38]. Yet, as this analysis has showed, several elements of information have been omitted or were poorly described in the PIS/ICF of the 11 adRCTs assessed – which could be justified in some instances. In future trials and pandemics, investigators should elaborate in the trial protocol why one or more elements of information specified in the GCP and/or by the Declaration of Helsinki were incompletely described or omitted from the PIS/ICFs.

We believe there is no need to modify the current GCP and Declaration of Helsinki requirements for the elements of information included the PIS/ICFs of trials conducted in a pandemic. However, investigators should inform RECs on their reasons for omission or incomplete description in the trial protocol, allowing them to identify and deliberate on the appropriateness of the decisions and to suggest or ask for any further changes to the PIS/ICF. This approach will ensure that the PIS/ICFs are adequately adapted to the needs of the population from which trial participants will be drawn.

## Additional material


Online Supplementary Document
