# Supplementary material for: Adaptive, platform trials assessing therapies for hospitalized COVID-19 patients: Informed consent forms omitted a few important elements of information
Source: J Glob Health. 2023 May 12;13:06019. doi: 10.7189/jogh.13.06019 (PMC10173897; doi:10.7189/jogh.13.06019)
Supplement: Online Supplementary Document [file jogh-13-06019-s001.pdf]

Supplementary document

Rafael Dal-Ré, Teck Chuan Voo, and Søren Holm

	Page
Supplementary document–Table S1	2
Supplementary document–Table S2	4
Supplementary document–Table S3	6
Supplementary document–Table S4	10

1.- Supplementary document–Table S1

The search comprised three stages:

1.-Cochrane Covid-19 Study register*. The search comprised the following descriptors: Adaptive/Platform trials; date: from 1 January 2020 to 28 March 2022. This search found 71 entries of 68 trials

* Data sources for Cochrane's COVID-19 Study Register include: PubMed, Embase, Cochrane Central Register of Controlled Trials (CENTRAL), ClinicalTrials.gov, WHO International Clinical Trials Registry Platform (ICTRP) and medRxiv. ClinicalTrials.gov is searched daily (Monday-Friday); PubMed, Embase, ICTRP and medRxiv weekly; and CENTRAL monthly (after CENTRAL publishes at the end of each month). (link: https://community.cochrane.org/about-covid-19-study-register).

HOSPITALIZED	NCT04315948 (DISCOVERY); NCT02735707 (REMAP–CAP); NCT04593940 (ACTIV–1 IM); NCT04501978 (ACTIV–3); NCT04924660 (ACTIV–4 HOST TISSUE); NCT04583956 (ACTIV–5–BET–A); NCT04583969 (ACTIV–5–BET–B); NCT04988035 (ACTIV–5 –BET–C); NCT04891133 (EU SOLIDACT); 2020-001736-95 / ISRCTN57085639 (ACCORD); IRCT20211221053470N1 / ISRCTN18066414 (SOLIDARITY PLUS); ISRCTN40580903 (CATALYST); NCT04390464 (TACTIC–R) NCT04381936 (RECOVERY); NCT04801940 / ISRCTN15851697 (HEAL–COVID)
EXCLUDED	
NO RESULTS, NO PUBLICATION NO PROTOCOL, NO INFORMED CONSENT FORM	NCT04345289 (CCAP); NCT04483960 (ASCOT–ADAPT); NCT04393246 (TACTIC–E); NCT04351724 (ACOVACT); NCT04386070 (PROTECT–SURG); NCT04330690 (CATCO); NCT04575064 (SOLIDARITY GERMANY);
OUTPATIENTS	ACTRN12620000566932; EUCTR2020-001528-32 (ARCO–HOMESTUDY); ISRCTN86534580 (PRINCIPLE); NCT04662086 (COPPS); NCT04662073 (COPPS); NCT04662060 (COPPS); NCT04575064; NCT04354428; PACTR202006537901307 (ANTICOV); PACTR202007700757139 (TOGETHER); NCT04885530 (ACTIV–6); ISRCTN30448031; CTRI/2022/01/039235; NCT04790786 (OPTIMISE–C19); NCT04401579 (ACTT–2);
SUSPENDED/PREMATURELY ENDED	NCT04341870 (CORUMINO–VIRO); EUCTR2020-001243-15-BE;
NO PLATFORM PHASE 2 & 3 RCTS	NCT04476979 (TOCIDEX); NCT04345991 (CORIPLASM); NCT04344756 (CORIMMUNO–COAG); NCT04324073 (CORIMUNO–SARI); NCT04343144 (CORUMINO–NIVO); ACTRN12620000557932 (ALLIANCE–TURKEY); NCT04355143 (COLHEART–19); NCT04826588 (SWISSPED–RECOVERY, N=75); NCT04346797 (CORUMINO19–ECU); NCT04344782 (CORIMMUNO–BEVA); NCT04359095; EUCTR2020-001614-38-BE; EUCTR2020-001860-27, NCT04746183 (AGILE); NCT04445467 (VIRCO); NCT04370262; 2020-001739-28 (DAWN–ANTICO); NCT04822818 (BEVA);
INDUSTRY-SPONSORED	NCT04460183; NCT04590586; NCT05077332; NCT04629703; NCT04940182
VACCINES	EUCTR2021-005197-25-DE; NCT04333732 (CROWN CORONA); NCT05037188
OTHER	NCT04324047; ISRCTN14226970; NCT04561063; NCT04498273; NCT04703608 (PATS–COVID)

2.-Clinicaltrials.gov. The search comprised the following descriptors: Date: 28 March 2022; Condition: covid–19; Other terms: adaptive; Study type: interventional studies (clinical trials); Funder type: NIH, Other US Federal agency and All others (individuals, universities, organizations); Study results: all studies; Study documents: study protocols and Informed consent. This search found 11 trials: 10 were excluded (3 for being conducted in outpatients, and 7 for not being platform trials), that allows us to add one new trial to the list, ITAC.

Trials excluded: ACTT-1, ACTT-2, ACTT-3, ACTT-4, NCT04498273, NCT04429854, NCT04354428, NCT04343989, ACTIV-2, CAPRI

3a.-From Vanderbeek et al study (Contemp Clin Trials 2022, 112: 106625) the following adaptive, platform trials fulfilling our requirements were identified: ACTIV (1 IM, 3, 4A, 4HT & 5), CATALYST, Discovery, EU-SolidAct, RECOVERY, REMAP-COVID, Solidarity and TACTIC-R. Solidarity, that was not identified with the previous searches, was added to the list.

3b.- From Noor et al (BMJ Open 2022, 12:e055615) the following adaptive platform trials fulfilling our requirements were identified: ACTIV (1 IM, 3, 4A), HEAL-COVID, RECOVERY, Solidarity and TACTIC-R. No new one could be added to the list

2.- Supplementary document–Table S2

Elements of informed consent

Declaration of Helsinki**.** 64th WMA General Assembly, Fortaleza, Brazil, October 2013.

(https://www.wma.net/policies-post/wma-declaration-of-helsinki-ethical-principles-for-medical-research-involving-human-subjects/)

Informed consent

26. In medical research involving human subjects capable of giving informed consent, each potential subject must be adequately informed of the aims, methods, sources of funding, any possible conflicts of interest, institutional affiliations of the researcher, the anticipated benefits and potential risks of the study and the discomfort it may entail, post-study provisions and any other relevant aspects of the study. The potential subject must be informed of the right to refuse to participate in the study or to withdraw consent to participate at any time without reprisal. Special attention should be given to the specific information needs of individual potential subjects as well as to the methods used to deliver the information.

After ensuring that the potential subject has understood the information, the physician or another appropriately qualified individual must then seek the potential subject's freely-given nformed consent, preferably in writing. If the consent cannot be expressed in writing, the non-written consent must be formally documented and witnessed.

All medical research subjects should be given the option of being informed about the general outcome and results of the study.

Good clinical practice guidelines. (https://ichgcp.net/4-investigator)

4. Investigator

4.8 Informed Consent of Trial Subjects**.**

4.8.10 Both the informed consent discussion and the written informed consent form and any other written information to be provided to subjects should include explanations of the following:

(a) That the trial involves research.

(b) The purpose of the trial.

(c) The trial treatment(s) and the probability for random assignment to each treatment.

(d) The trial procedures to be followed, including all invasive procedures.

(e) The subject's responsibilities.

(f) Those aspects of the trial that are experimental.

(g) The reasonably foreseeable risks or inconveniences to the subject and, when applicable, to an embryo, fetus, or nursing infant.

(h) The reasonably expected benefits. When there is no intended clinical benefit to the subject, the subject should be made aware of this.

(i) The alternative procedure(s) or course(s) of treatment that may be available to the subject, and their important potential benefits and risks.

(j) The compensation and/or treatment available to the subject in the event of trial-related injury.

(k) The anticipated prorated payment, if any, to the subject for participating in the trial.

(l) The anticipated expenses, if any, to the subject for participating in the trial.

(m) That the subject's participation in the trial is voluntary and that the subject may refuse to participate or withdraw from the trial, at any time, without penalty or loss of benefits to which the subject is otherwise entitled.

(n) That the monitor(s), the auditor(s), the IRB/IEC, and the regulatory authority(ies) will be granted direct access to the subject's original medical records for verification of clinical trial procedures and/or data, without violating the confidentiality of the subject, to the extent permitted by the applicable laws and regulations and that, by signing a written informed consent form, the subject or the subject's legally acceptable representative is authorizing such access.

(o) That records identifying the subject will be kept confidential and, to the extent permitted by the applicable laws and/or regulations, will not be made publicly available. If the results of the trial are published, the subject’s identity will remain confidential.

(p) That the subject or the subject's legally acceptable representative will be informed in a timely manner if information becomes available that may be relevant to the subject's willingness to continue participation in the trial.

(q) The person(s) to contact for further information regarding the trial and the rights of trial subjects, and whom to contact in the event of trial-related injury.

(r) The foreseeable circumstances and/or reasons under which the subject's participation in the trial may be terminated.

(s) The expected duration of the subject's participation in the trial.

(t) The approximate number of subjects involved in the trial.

3.- Supplementary document–Table S3

Conflicts of interest (CoIs) with industry disclosed by authors in the articles

1.BARI-SOLID ACT, Critical Care 2023, 27: 9

MT has been member of scientific advisory board for Lilly.
JRA has received advisory fees from Lilly.
JP reports lecture fees from Gilead; support for attending meetings from Gilead, Eumedica, Merck Sharp & Dohme, outside the submitted work.
ARH reports personal fee from Pfizer (2021) for lectures outside the submitted work.
MH(it) has received personal fees from Gilead (2020) and Pfizer (2021) for editing and lectures outside the submitted work, and travel/congress grants from Pfizer (2020, 2021), and Gilead (2022).
MJ reports consulting or speakers fees from Baxter, Gilead, CLS Behring, AM-Pharma, Novartis, Fresenius and grant support from Fresenius, Baxter, outside the submitted work. JAP reports fees for lectures and advisory boards from MSD, Pfizer, Astra-Zeneca, Jansen, Gilead, AOP Orphan Pharmaceuticals,
Cepheid MB reports an unrestricted grant for Moderna (2022) outside the submitted work.
MB reports an unrestricted grant for Moderna (2022) outside the submitted work.
KL reports personal fees from Gilead, MSD, Janssen and ViiV Healthcare for advisory boards and lectures outside of the submitted work.
JM reports personal fees from Pfizer (2017) for lectures outside the submitted work and travel fees from Pfizer (2022) and Menarini (2021).
JCR reports a grant from Hamilton medical (2019–2020) outside the submitted work.
FLJ reports Helse Sor-Ost grant for developing COVID-19 serology (2020–2021) and Grant from CEPI to monitor responses in patients (2021–2023).
DC reports an HIV grant from Janssen (2019–2020), personal fees from Gilead (2020) and Pfizer (2022) for lectures outside the submitted work.

2.CATALYST, Lancet Resp Med 2022, 10: 255-66

BAF reports consultancy for Novartis, Bristol Myers Squibb, Servier, Galapagos, and Janssen and research funding from Servier and Galapagos.
MR is currently undertaking a Senior Clinical Fellowship financed by Roche.
PK reports consultancy for Bristol Myers Squibb and AstraZeneca, and research funding from Bayer and Pfizer.

3.DISCOVERY, Clin Microbiol Infect 2021, 27: 1826e1837

FR reports personal fees from Gilead Sciences, personal fees from MSD, personal fees from Pfizer, personal fees from TheraTechnologies, personal fees from ViiV Healthcare, outside the submitted work.
FG reports grants from BioMerieux, personal fees and non-financial support from Gilead, non-financial support from Corevio, outside the submitted work.
GP reports grants and personal fees from Gilead Sciences, grants and personal fees from Merck, grants and personal fees from ViiV Healthcare, grants and personal fees from TheraTechnologies, outside the submitted work.

KL reports personal fees and non-financial support from Gilead, personal fees and non-financial support from Janssen, personal fees and nonfinancial support from MSD, personal fees and non-financial support from ViiV Healthcare, personal fees and non-financial support from Abbvie, during the conduct of the study.
YY has nothing to disclose. He has been a board member receiving consultancy fees from ABBVIE, BMS, Gilead, MSD, J&J, Pfizer, and ViiV Healthcare; however, all these activities have been stopped in the 3 past years.
FL reports personal fees from Gilead, personal fees and non-financial support from MSD, non-financial support from Astellas, and nonfinancial support from Eulmedica, outside the submitted work.
AK reports personal fees from Baxter, personal fees from Aspen, and personal fees from Aguettant, outside the submitted work.
SN reports personal fees from MSD, personal fees from Pfizer, personal fees from Gilead, personal fees from Biomerieux, and personal fees from BioRad, outside the submitted work.
FD reports personal fees from Gilead, outside the submitted work. JN reports non-financial support from MSD France, non-financial support from GILEAD Sciences and personal fees from PASCALEO, outside the submitted work.
JM reports non-financial support from GILEAD, outside the submitted work.
AM reports personal fees from MSD, personal fees from GILEAD, personal fees from JANSSEN and personal fees from Viiv Healthcare, outside the submittedwork.
MH reports grants from Fonds Erasmed COVID –Universite Libre de Bruxelles, grants from Belgian health Care Knowledge Centre, during the conduct of the study, personal fees from Gilead advisory board on education on invasive fungal infections, personal fees from Pfizer: moderator for session on Isavuconazole, outside the submitted work.
DC reports personal fees from Gilead, grants and personal fees from Janssen, outside the submitted work.
CB reports personal fees from Da Volterra and personal fees from Mylan Pharmaceuticals, outside the submitted work.
FM reports grants from Sanofi and grants and personal fees from Da Volterra, outside the submitted work.
All other authors have nothing to disclose.

4.ITAC, Lancet 2022, 399: 530-40

MNP reports grants from University of Minnesota (Minneapolis, MN, USA) during the conduct of the study, grants from National Institutes of Health (NIH) during the conduct of the study, Gilead Sciences, ViiV, Celgene, and Janssen Pharmaceuticals outside the submitted work.
AGB reports grants from University of Minnesota during the conduct of the study, grants from UK Research and Innovation (UKRI) outside of the submitted work.
MKJ reports donation of trial medications from Regeneron Pharmaceuticals, Janssen Pharmaceuticals, and from Merck; and grants, personal fees, and donation of trial medications from Gilead Sciences, outside the submitted work.
SLP reports grants from University of Minnesota during the conduct of the study, European and Developing Countries Clinical Trials Partnership, UKRI, Academy of Medical Sciences, ViiV Healthcare, Medical Research Council, and Gilead Sciences outside the submitted work.
MKD reports being an employee of CSL Behring.
SP reports being an employee of CSL Behring.
CH reports being an employee of Emergent;
Ramanathan reports being an employee of Emergent.
HC reports being an employee of Gilead Sciences.
EM reports being an employee of Grifols.
TW reports being an employee of Grifols.

JVT reports being an employee of Takeda.
LY reports being an employee of Takeda.
JDN reports grants from NIH during the conduct of the study.
All other members of the writing group declare no competing interests.

5.RECOVERY, Data extracted from the CoIs forms, supplemental information of the article: N Engl J Med 2021, 384: 693-704. Only those authors with CoIs with industry are listed.

Dr. Bell reports grants from UKRI/NIHR, non-financial support from Roche, during the conduct of the study;
Dr. Emberson reports grants from UKRI/NIHR, non-financial support from Roche, during the conduct of the study; grants from Boehringer Ingleheim, outside the submitted work;
Dr. Faust reports grants and other from Pfizer, other from AstraZeneca/MedImmune, grants and other from Sanofi, grants and other from Merck, other from Seqrius, other from Sandoz, grants from GSK, grants from J&J, outside the submitted work;
Dr. Haynes reports grants from UKRI/NIHR, non-financial support from Roche, during the conduct of the study; grants from The Medicines Company, grants from Boehringer-Ingelheim, outside the submitted work; .
Dr. Horby reports grants from UKRI/NIHR, non-financial support from Roche, during the conduct of the study;
Dr. Landray reports grants from UK Research and Innovation, grants from National Institute for Health Research, grants from Health Data Research UK, during the conduct of the study; grants from Novartis, grants from Boehringer Ingelheim, grants from Merck Sharp & Dohme, outside the submitted work;
Dr. Lim reports grants from National Institute for Health Research, grants from Pfizer, outside the submitted work; .
Dr. Linsell reports grants from UKRI/NIHR, non-financial support from Roche, during the conduct of the study; .
Dr. Mafham reports grants from UKRI/NIHR, non-financial support from Roche, during the conduct of the study; grants and non-financial support from The Medicines Company/Novartis, outside the submitted work;
Dr. Staplin reports grants from UKRI/NIHR, non-financial support from Roche, during the conduct of the study; grants from Boehringer-Ingelheim, outside the submitted work.

6.REMAP–COVID, Jama 2020, 324: 1317-29

Dr Angus reported receiving personal fees from Ferring Pharmaceuticals Inc, Bristol-Myers Squibb, Bayer AG, and ALung Technologies Inc outside the submitted work;
Dr Bradbury reported receiving personal fees from Bristol-Myers Squibb, Pfizer, Janssen, Amgen, Novartis, Portola, Bayer, and Ablynx outside the submitted work.
Dr Buxton reported receiving grants from the Breast Cancer Research Foundation during the conduct of the study and grants from Bayer, Amgen, Eli Lilly and Company, Janssen, Kazia Therapeutics, DelMar Pharma, Eisai, the National Brain Tumor Society, the National Foundation for Cancer Research, and the Asian Foundation for Cancer Research; gifts from the Yousefzadeh Family Foundation and Jeffrey Tarrant; and personal fees from Berry Consultants LLC outside the submitted work.
Dr de Jong reported receiving personal fees from Roche, Janssen, Vertex, and Visterra outside the submitted work.
Dr Gordon reported receiving grants from the NIHR and the NIHR Research Professorship; nonfinancial support from the NIHR Clinical Research Network and the NIHR Imperial

Biomedical Research Centre during the conduct of the study; and personal fees from GlaxoSmithKline and Bristol-Myers Squibb outside the submitted work.
DrMcAuley reported receiving personal fees from GlaxoSmithKline, Boehringer Ingelheim, and Bayer for consultancy outside the submitted work;
Dr McVerry reported receiving salary support from UPMC Learning While Doing Program and the Translational Breast Cancer Research Foundation during the conduct of the study and grants from Bayer Pharmaceuticals Inc and the NIH/National Heart, Lung, and Blood Institute outside the submitted work.
Dr Seymour reported receiving grants from the NIH's National Institute of General Medical Sciences and personal fees from Beckman Coulter Inc and Edwards Lifesciences Inc outside the submitted work.

7.Solidarity, Data extracted from the CoIs forms, supplemental information of the article: N Engl J Med 2021, 384: 497-511. Only those authors with CoIs with industry are listed.

Dr. Alvarez-Moreno reports personal fees from Gilead, grants from Pfizer, personal fees from MSD, personal fees from Sanofi, outside the submitted work.

Dr. Jancoriene reports personal fees and non-financial support from Merck Sharp & Dohme, personal fees and non-financial support from AbbVie, personal fees and non-financial support from Pfizer, personal fees and non-financial support from Sanofi, outside the submitted work;

Dr. Lopardo reports personal fees from Gilead, outside the submitted work;
Dr. Manuel reports personal fees from Syneos, grants from Lophius, personal fees from Gilead, outside the submitted work;
Dr. Nunes reports grants from PFizer, during the conduct of the study; personal fees from Gilead Sciences, personal fees from Abbvie, outside the submitted work;

4.- Supplementary document–Table S4

RECOVERY trial.

Participant's information sheet/informed consent form V4.1 16 April 2020.

The information regarding adverse reactions associated to the medicinal products to be assessed was the following:

"The treatments, which may be given in addition to the usual care at your hospital, are: Lopinavir-Ritonavir (commonly used to treat HIV); corticosteroids (a type of steroid, which are used in a range of conditions typically to reduce inflammation [the precise type differing in pregnant women and other participants, but all in common use]); hydroxychloroquine (a treatment for malaria); or azithromycin (a commonly-used antibiotic). For patients whose condition is more severe, tocilizumab (a treatment for rheumatoid arthritis) is also an option. At present, we don't know whether any of these are effective in treating COVID-19. However, the side-effects are well-known from other uses and your doctor will be able to monitor you appropriately.

In addition, when referring to the possible risks to be known by participants:
<u>7) What are the possible risks of being in the study?</u>
Apart from the known side effects of these treatments (which may include tummy upset, 'flu-like symptoms, and blood test abnormalities), there is the unlikely possibility of a severe reaction to a study drug. Although Tocilizumab has been very rarely associated with liver damage in prolonged use this is not expected to be a problem with the short-term administration in this study. Women who are pregnant may be included, however, the effect of some of the treatments on unborn babies is uncertain - although all the treatments have previously been used in pregnancy for other medical conditions without safety concerns being raised. If you do receive treatment and are not already pregnant, as a precaution, we advise that you should not get pregnant within 3 months of the completion of the trial treatment(s). Please ask your hospital doctor if you would like more information. Once you have been included in the study, you and your doctors will know which treatment the computer has allocated for you. Your doctors will be aware of whether there are any particular side effects that they should look out for.

This approach has been modified. The last PIS/ICF available (V22.0, 5 Mar 2022),
"The treatments for COVID-19, which may be given in addition to the usual care at your hospital, include a high dose steroid, dexamethasone (if you have low oxygen levels), a treatment for diabetes or heart failure called empagliflozin, a synthetic antibody treatment directed against the virus (called sotrovimab) and two antiviral treatments called molnupiravir and Paxlovid.
The treatments for influenza pneumonia, which may be given on top of your usual care, include two anti-viral treatments, oseltamivir and baloxavir and low-dose dexamethasone. At present, we don't know whether any of these will work. However, the side-effects are already well-known from other uses and so your doctor will be able to monitor you appropriately.

In addition, when referring to the possible risks to be known by participants:
<u>7) What are the possible risks of being in the study?</u>
– Dexamethasone may also disturb sleep and increase the risk of infections. In people with diabetes it can raise blood sugar.

– Empagliflozin may cause urine or genital tract infections, like thrush. If you have diabetes, empagliflozin also lowers blood sugar in people taking insulin or some other diabetes treatments so your doctors may adjust the doses of those. It may also cause a condition called ketoacidosis (which rarely can be life-threatening), which is treated with a drip and insulin. You will be monitored for this with daily fingerprick blood or urine tests.

– Oseltamivir may cause headache, tummy upset and allergic reactions.

– Baloxavir rarely causes allergic reactions, but has no other known side effects.

– Sotrovimab is given by intravenous infusion and may cause allergic reactions during the infusion, but severe reactions have been rare.

– Molnupiravir may cause dizziness, headache, tummy upset and rashes.

– Paxlovid may cause altered taste and tummy upset.

There is also the unlikely possibility of a severe reaction to any study drug. Please ask your hospital doctor if you would like more information. Once you have been included in the study, you and your doctors will know which treatment the computer has allocated for you. Your doctors will be aware of whether there are any particular side effects that they should look out for.

Women taking molnupiravir or Paxlovid should not get pregnant while taking the drug or for 4 days afterwards. Women using the combined oral contraceptive who receive Paxlovid should use either additional barrier contraception or an alternative effective method until after one complete menstrual cycle after leaving hospital. Women who are pregnant may be included, however, the effect of some of the treatments on unborn babies is uncertain. Pregnant women will not receive empagliflozin, Paxlovid (in first 12 weeks of pregnancy) or molnupiravir as it may be harmful in pregnancy or when breast-feeding. Dexamethasone and oseltamivir have previously been used in pregnancy for other medical conditions without safety concerns being raised. Baloxavir and sotrovimab (and Paxlovid after 12 weeks of pregnancy) have not been used in pregnant women before but are considered to have an acceptably low level of risk to use in pregnant women in this trial by a national expert panel; your medical team will discuss with you whether you would be willing to receive them.
